# Typology of civil engineers’ mental model about artificial intelligence

**DOI:** 10.3389/frai.2026.1812314

**Published:** 2026-07-01

**Authors:** Ardalan Feili, Masih Rajabi Shabankareh, Sima Alipour, Shahrzad Entezaralmahdi, Shahryar Sorooshian

**Affiliations:** 1 Department of Management, Apadana Higher Education Institute, Shiraz, Iran; 2 Department of Civil Engineering, Apadana Higher Education Institute, Shiraz, Iran; 3 Department of Civil Engineering, University of Texas at Arlington, Arlington, TX, United States; 4 Department of Business Administration, University of Gothenburg, Gothenburg, Sweden; 5 Faculty of Engineering and Sustainable Development, University of Gävle, Gävle, Sweden

**Keywords:** artificial intelligence, behavior, civil engineers, mental models, Q methodology

## Abstract

**Purpose:**

This study aims to identify, classify, and typologize the mental models of civil engineers, the diversity of their perspectives, and their intellectual structures regarding the integration of artificial intelligence in the industry.

**Method:**

The philosophical framework of this research is based on an interpretive–positivist paradigm (Q method) and its application. The 11 participants were selected from individuals with sufficient knowledge, expertise, and experience related to the research topic, and who could provide the necessary information to achieve the study’s objectives. The purpose of this sampling is to classify them and identify common mental patterns within a small and targeted group, rather than to generalize the findings to a larger population.

**Findings:**

The results indicate that civil engineers’ mental models regarding the applications of artificial intelligence are divided into three categories: hopeful-concerned, black-box thinkers, and results-oriented.

**Conclusion:**

Analyzing the distinct thought patterns among civil engineers reveals significant heterogeneity in the acceptance of AI, highlighting the need for fundamental changes in communication strategies. The study shows that to facilitate use, key information must be delivered through tailored content. For example, for the black-box group, content should emphasize algorithmic transparency and how models work. For the hopeful-concerned group, content should reduce ethical and employment-related concerns. Meanwhile, for the results-oriented group, content should highlight economic returns and proven successes. Ultimately, this approach is essential for effective and successful integration of AI into the infrastructure industry.

## 1 Introduction

The construction industry is a major contributor to economic development, yet it is also known for its complexity, high risk, and relatively low productivity. These characteristics have created a strong need for digital transformation (Pan and Zhang, 2021). Artificial intelligence and its subfields are among the most influential technologies in this transformation, causing to increase in productivity, reducing costs, improving safety, and elevating the quality of infrastructure across the entire lifecycle of civil engineering projects. It spans the entire lifecycle of civil engineering projects from planning and design to operation and maintenance (Abioye et al., 2021). Applications of AI range from predicting problems in scheduling and traffic optimization to detecting failures in structures (Khan et al., 2025). Because of these benefits, AI is considered a powerful tool that will increasingly become an essential component of civil engineering (Kapoor et al., 2024). Despite this promise, the adoption and integration artificial intelligence into traditional and dynamic environments, such as construction sites, face significant challenges (Pan and Zhang, 2021). Most of these challenges stem from human and organizational factors, including ethical issues, lack of technical expertise within the workforce, and high implementation costs (Sira, 2025). Another key barrier is the explainability of AI models, which makes it difficult for engineers to interpret results. This barrier affects trust and acceptance of the technology (Love et al., 2023). These issues highlight that technological development is insufficient, while understanding how professionals think and judge is critical (Pasquale, 2019).

However, most of the existing research has focused on the technical capabilities of AI; less attention has been paid to the diverse perspectives among engineers (Brown and Vergragt, 2008). The civil engineering industry faces complex challenges in adopting AI. These challenges go beyond financial or technical barriers (Sargiotis, 2024a). One major barrier is the need for multidisciplinary expertise as effective use of AI demands combining complex civil engineering knowledge (including structural, geotechnical, and project management) with new skills in data science and machine learning. Moreover, recent studies show that employees’ perspectives directly influence technology adoption within organizations. When AI is perceived as a radical innovation—one that leads to fundamental changes and creates uncertainty—there is more resistance to being viewed as an incremental innovation (Greve and Seidel, 2026). This cultural and organizational resistance often happens due to the shortage of specialized personnel and the high cost of implementing AI tools (Aziz Baker, 2025). Accordingly, it has been revealed that individuals’ motivation to change behavior, rather than organizational capabilities alone, is a key factor in the implementation of advanced technologies (Huang, 2023).

Therefore, examining how these perspectives are formed is a prerequisite for successful adoption of artificial intelligence. Traditional research tools, like quantitative surveys using Likert scales, are unable to fully reveal the deeper layers of viewpoints among professionals (Ramlo and Newman, 2011). These methods produce a single or averaged viewpoint. It ignores the diversity of opinions that exists among individuals (McKeown and Thomas, 2013; Brown, 1980). Technical or economic studies cannot fully explain the gap between AI capabilities and engineers’ willingness to adopt it. This gap reflects a set of subjective interpretations and personal judgments shaped by the diverse roles and professional experiences of civil engineers (Dreksler et al., 2025). For this reason, studies seek to understand more deeply the factors of accepting technology require methodologies that focus on active subjectivity (Stephenson, 1953). Q methodology is specifically designed to address this need. By combining qualitative and quantitative approaches, it enables the discovery and definition of common thought patterns (Calvo and Peters, 2014; Dziopa and Ahern, 2011). This methodology uses professionals with similar viewpoints to understand which factors, such as job-related risk, technical ambiguity, or economic potential, carry greater importance for each group. By concentrating on individuals’ internal interpretations and perceptions of reality, rather than relying on external variables, Q methodology uncovers the distinct cognitive patterns that shape motivations and resistance (Watts and Stenner, 2012).

Given the increasing role of artificial intelligence in the construction industry and the development of its use in construction projects, this study aimed to typify the mindsets of civil engineers about artificial intelligence to facilitate planning for the adoption and use of artificial intelligence in the construction industry. A literature review shows that in developing countries, and especially in the Middle East (given the similarity of their cultural and economic environments), few studies have been conducted in this area, so this study helps to fill the research gap.

## 2 Theoretical background of the research

This section reviews the theoretical foundations related to the research topic. First, the applications of artificial intelligence in civil engineering and its transformative potential in the areas of design, project management, and infrastructure maintenance are reviewed. Then, theoretical frameworks of technology acceptance, especially the Technology Acceptance Model (TAM) and the Theory of Planned Behavior, are explained. Next, factors affecting the acceptance of artificial intelligence by civil engineers, including perceived usefulness, ease of use, social norms, cognitive, organizational, and technical barriers, are discussed. The purpose of this section is to provide the necessary theoretical background to better understand the acceptance of artificial intelligence in the civil engineering industry and identify gaps in the theoretical literature.

### 2.1 Artificial intelligence for civil engineers

Artificial intelligence, as a key technology, has great potential to drive fundamental changes across all subfields of civil engineering (Sargiotis, 2024b). The applications of AI in this field can be categorized into three major areas: enhanced decision-making, increased productivity, and intelligent infrastructure monitoring (Khan et al., 2024). In structural design, genetic algorithms and reinforcement learning are used to identify optimal design solutions with minimal material consumption and maximum environmental sustainability (Almutairi, 2024). These intelligent systems can evaluate thousands of design scenarios in a short period. In project management and construction, neural networks are used for risk analysis and accurate predictions of schedule and cost delays (Demiss and Elsaigh, 2024). These predictions help managers to take corrective actions before the problems happen. Additionally, in asset management and infrastructure maintenance, such as bridges, tunnels, and dams, AI can detect potential failures much earlier than traditional methods by using sensors (Plevris and Papazafeiropoulos, 2024). These widespread applications of artificial intelligence have made this tool essential for enhancing the quality and sustainability of civil engineering projects (Bahadori-Jahromi et al., 2025). As noted earlier, AI has become a transformative force across the entire construction industry. In risk management, deep learning models reduce the likelihood of high-risk events such as budget overruns or schedule delays (Wu and Zhou, 2023).

Technology adoption is defined as the psychological and behavioral process by which individuals or organizations begin and continue to use an innovation (Rogers, 2003). Several theoretical methods have been developed to explain this process, the most famous of which include the Technology Acceptance Model and the Theory of Planned Behavior. Based on these models, two primary factors determine the adoption of new technology: perceived usefulness and perceived ease of use (Venkatesh et al., 2003). Perceived usefulness belief that using the technology will improve job performance (Lozano-Ramírez et al., 2023), whereas perceived ease of use reflects the level of effort required to learn and work with that technology (Dey Martino and Brunner, 2024).

However, environmental and social factors play a critical role in professional environments. Social norms, such as the influence of colleagues and managers on individual decision-making, and facilitating conditions, including technical support and the availability of necessary infrastructure, significantly shape behavioral intention (Vannoy and Palvia, 2010). In engineering industries, an organizational culture that focuses on accuracy, safety, and proven methodologies can affect the level of acceptance for risky or novel technologies. Therefore, assessing the adoption of emerging technologies such as artificial intelligence requires understanding how engineers interpret and evaluate these factors (Mühlroth and Grottke, 2020).

The adoption of artificial intelligence in the civil engineering sector faces challenges that are often individual, cognitive, and organizational in nature (Soomro et al., 2026). One of the most significant barriers is the perception of loss of freedom and control. Engineers worry that excessive reliance on automated outputs may reduce their judgment skills and experiential expertise, elements deeply tied to their professional identity (Tomori and Ogunseiju, 2024). This concern is directly related to how engineers value their implicit knowledge compared with automated systems.

Nevertheless, widespread adoption of these tools encounters significant organizational and technical barriers. One major challenge is data quality and standardization, because AI models require large volumes of clean and relevant data in civil engineering, while project environments often face irregular, incomplete, and fragmented datasets (Narayanan, 2025). Moreover, although AI algorithms can produce accurate predictions, accountability and responsibility in the event of errors create a serious legal and ethical dilemma for engineers. This dilemma slows the adoption of technologies such as fully autonomous construction, as engineers cannot fully trust a “black box” system (Winfield and Jirotka, 2017). Another major organizational barrier that delays adoption is cultural resistance to high initial investments and the perception of AI as a threat to existing jobs (Shang et al., 2023).

In addition, trust in algorithms is a very important mental factor (Alexander et al., 2018). Engineers are hesitant to fully adopt AI systems unless they trust the transparency or explain ability of the models (Zhang et al., 2024). Another challenge is the fear of replacing human labor, which creates a strong mental factor in resisting AI adoption (Kowal et al., 2025). This fear, rooted in perceived job security, is a behavioral concern that cannot be addressed through quantitative models alone. Therefore, understanding the diverse mental models, the most essential step in designing effective educational and organizational strategies is to address these challenges, like the tension between valuing tacit knowledge and accepting automation (Li et al., 2022).

### 2.2 Experimental background

Krampell et al. (2020), in a study aimed at identifying and analyzing distinct mental models with the focus on “smart construction,” discovered three key patterns: a group that emphasizes on efficiency and economic gain, a group worried about the loss of traditional skills, and a third group that viewed practical implementation barriers and weak data infrastructures as the primary challenges. Uddin (2025), who aimed to evaluate organizational and cultural factors influencing AI adoption in project management firms, observed that the most critical factor in overcoming employee resistance is active support from senior leadership in fostering an innovative culture. From the perspective of younger professionals, Martin et al. (2025) aimed to analyze the views of engineers under 30 about their career futures. They identified two major mental models: one group believed AI would be an opportunity for data-driven specialization, while another was concerned about the loss of the traditional identity of civil engineers. Zerilli et al. (2022), aiming to identify mental models of managers and clients regarding the transparency of predictive models, discovered two main patterns: one group cared just about the accuracy of final results, while the other considered insight into the algorithm’s internal reasoning essential for trust. In the safety, Wamba-Taguimdje et al. (2020), who investigated the effect of safety culture on engineers’ motivation to use AI-based monitoring systems, found that in organizations with strong safety cultures, engineers viewed these systems as supportive tools, whereas in weaker situations, the same systems were perceived as punitive surveillance mechanisms, leading to high resistance. Ghahari et al. (2024), examining barriers to implementing AI during the pre-construction and design phases, concluded that the biggest obstacle is not algorithmic flaws, but the lack of alignment in input data formats across different software, which reduces trust in full automation.

Finally, Singh et al. (2024), aiming to identify mental models of sustainability experts regarding the use of reinforcement learning in green building design, observed that while one major group embraced AI for its ability to rapidly process complex variables, another group exercised caution due to concerns about potential conflicts with local building standards.

Additionally, several experimental studies have examined the reasons behind the adoption of AI technologies by managers and civil engineers in management roles (Shang et al., 2023). These studies often focus on variables such as perceived competitive advantage and organizational adaptability (Zeraati Foukolaei, 2025). For example, King (2025), seeking to analyze the determinants guiding decision-making among executives in large construction companies, found that expectations of rapid investment return and competitive market pressure are the strongest drivers of AI adoption at the organizational level. This managerial view, primarily centered on profit and productivity, often differs from the perspectives of technical experts, who tend to prioritize accuracy, safety, and tacit knowledge (Chen et al., 2025). Meanwhile, Ma, Sun, and Chen et al. (2025) showed that managers who perceive cultural flexibility and strong learning capacity are more willing to accept the risks associated with adopting complex technologies such as AI. There is no unified viewpoint on AI based on these contrasts in adoption drivers across organizational levels, specifically between managers and technical staff (Huang et al., 2022a). Overall, experimental studies show that reasons for using and accepting AI are largely concentrated in information and communication technologies. Where variables such as perceived usefulness and easy impact on business processes are most prominent (Wamba-Taguimdje et al., 2020). However, in civil engineering, these main reasons for acceptance are moderated by additional technical and non-technical concerns such as explain ability, safety, and the preservation of engineering judgment (Hayes et al., 2021). Accordingly, the importance of classifying civil engineers’ mental models for both managerial and technical roles has been experimentally established as essential for accurately identifying distinct and conflicting viewpoints and for designing strategies.

## 3 Research methodology

The philosophical framework of this study is the interpretive–positivist paradigm through the Q-methodology. Q-methodology is a technique that enables researchers, first, to identify and categorize individual beliefs, and second, to classify groups of individuals based on these perceptions (McKeown and Thomas, 2013). The primary purpose of this technique is to reveal different patterns of thought rather than to count how many people hold each type of thinking.

As described below, this study employs the five-step Q-methodology process (Brown et al., 1999):

Collecting the Concourse: All information, opinions, and perspectives about the research topic are gathered from various sources (e.g., interviews, notes, articles, and other materials). Preparing the Q-Sample: By evaluating the content of the concourse, essential statements are extracted. These statements are rewritten as short, independent phrases, and a Q-sample is selected. Each Brown statement is printed on a separate card to form the deck of Q-cards. Selecting Participants: Individuals whose views and opinions on the research topic are to be examined are selected. Sorting: Participants sort the Q-cards based on specific instructions. This stage constitutes the data-collection process. Data Analysis: The collected data are analyzed using factor-analytic techniques, and the extracted factors are then interpreted.

This study is considered applied research. Its main objective is to identify and describe the mental models and perspectives of civil engineers regarding the applications of artificial intelligence. A non-probability, purposive sampling strategy was used to select participants. In this approach, the researcher selects individuals who possess sufficient knowledge, expertise, and experience related to the research topic and who can provide the relevant information necessary to achieve the study’s goals. The aim of this type of sampling is not generalization to a larger population, but rather capturing the full range of possible viewpoints for classification. Q-methodology studies typically involve sample sizes ranging from 8 to 40 participants (Watts and Stenner, 2012); One of the main strengths of the Q method is its reproducibility and that it provides statistically significant results from a relatively small sample size (Brown et al., 1999; Doody et al., 2009). Q participants are selected to represent a wide range of perspectives within a target population (Webler et al., 2009). The participants themselves are the key variables, so a large sample size is not necessary (Watts and Stenner, 2012). Typically, a Q study will result in 2 to 5 social perspectives. For each perspective, it is sufficient to have four to six people “defining” that perspective. According to this criterion, the number of Q participants should be between eight (2 factors × 4 people to define each factor) and 30 (5 factors × 6 people to define each factor). On the other hand, it is important that the number of Q participants is less than the number of Q statements. A ratio of 3 to 1 is usually used. For a study with 45 Q statements, the ideal number of Q participants would be 15 (Webler et al., 2009). Based on the two criteria presented, the number of participants in the current study (11 people) is acceptable, covering one-third of the 31 Q statements. In addition, the strict criteria for entering participants into the study, especially on the one hand, and the novelty of the application of artificial intelligence in the Iranian manufacturing industry on the other, limited the number of participants.

The inclusion criteria for study participants were purposefully defined to ensure sufficient professional expertise and relevance of participants’ experiences to the research topic. Accordingly, eligible participants were, first, required to hold a university degree in one of the subfields of civil engineering (at least at the bachelor’s level) or to have continuous professional employment in civil engineering–related positions (such as design, construction, supervision, or project management); second, to possess a minimum of 3–5 years of practical experience in civil engineering projects, preferably in roles involving decision-making responsibilities or participation in project management and control; third, to demonstrate a reasonable level of familiarity with the concepts and applications of artificial intelligence, which could have been acquired through the use of intelligent tools or systems in projects, participation in relevant courses and workshops, or engagement in reading and research activities in this domain; and fourth, to exhibit willingness and sufficient ability to actively participate in in-depth interviews and to clearly articulate their professional experiences, attitudes, and mental representations regarding artificial intelligence and its applications in civil engineering. Applying these criteria enabled the selection of 11 participants whose professional and cognitive characteristics were well aligned with the aims of the study, thereby providing rich and credible qualitative data, which is reported in Table 1.

**Table 1:** Characteristics of research participants.

Participants	Researcher’s familiarity with AI	Management history (year)	Career history (year)	Education	Age (year)
P_1_	2 Articles	5	12	Master’s degree	38
P_2_	1 Research	8	11	Master’s degree	35
P_3_	3 Articles	11	18	Ph.D	40
P_4_	5 Articles	19	24	Ph.D	50
P_5_	1 Research	10	15	Master’s degree	41
P_6_	2 Articles	6	10	Master’s degree	36
P_7_	4 Articles	13	16	Ph.D	42
P_8_	3 Articles	14	17	Ph.D	48
P_9_	2 Research & 2 Articles	15	21	Ph.D	39
P_10_	2 Research	6	10	Ph.D	38
P_11_	3 Research	8	12	Master’s degree	42

The main research question of this study is: What are the typologies of civil engineers’ mental models regarding the applications of artificial intelligence by using the Q-method? The main discourse of the study was gathered from various sources (the theoretical literature). After evaluating and summarizing the discourse space, 31 Q-statements were selected from 85 statements. These cards were then provided to the participants.

Finally, the data obtained from the participants’ sorting were entered into SPSS version 26 to identify the mental model patterns of civil engineers with the help of Q-factor analysis. Discussions about reliability and validity refer to the results of an instrument, not to the instrument itself. Since the results are produced for a specific set of conditions, statements about reliability and validity must include a description of the nature of those conditions. Hence, the results of an instrument are not generally reliable, but only for specific conditions (Nazariadli et al., 2019). In the Q method, to assess validity, the researcher must ask himself whether the collected statements are comprehensive enough to reflect different mentalities. In this regard, content validity was confirmed based on the ratings that participants gave to the statements and adjacent statements. To calculate the reliability coefficient of Q-sorting, the test–retest method was also used on 2 of the sample individuals. The subjects repeated the Q-sortings at intervals of one and two weeks. It was found that the subjects sorted the sentences in the same way and the correlation coefficients between the corresponding Q-sortings were higher than 0.75, as presented in Table 2.

**Table 2:** Test–retest reliability test results.

Participant ID	Interval	Correlation coefficient	Interpretation
P_3_	1 week	0.80	High reliability
P_3_	2 week	0.78	High reliability
P_7_	1 week	0.82	High reliability
P_7_	2 week	0.76	Acceptable reliability

### 3.1 Research findings

Factor analysis of the Q-methodology data identified three mental patterns (factors) that together explained 66.713% of the total variance. Each factor represents a distinct and meaningful viewpoint within the sample population. These mental patterns are as follows:

- Mental Pattern 1: The Worried-Hopeful Mindset
- Mental Pattern 2: The Black-Box Mindset
- Mental Pattern 3: The Result-Oriented Mindset

### 3.2 Formation of the Q-sort set and sorting procedure

After evaluating and organizing the discourse space, the final set of Q-statements was shown in Table 2. Participants can place the 31 Q-statements on a predefined scale (ranging from 1 to 9), placing as many cards as they wish at each level of the scale.

## 4 Q factor analysis

The correlation matrix was used to identify the mental patterns of civil engineers, which is a standard and commonly used method in factor analysis. The factors were rotated using the Varimax method. The values extracted from the Q factor analysis are presented in Table 3.

Based on the results presented in Table 4, three mental patterns (factors) were identified from the participants’ viewpoints, which together explain 66.713% of the total variance. The first mental pattern accounts for 35.213%, the second for 19.835%, and the third for 11.665% of the total variance. Table 5 presents the rotated factor matrix. According to this matrix, the participants associated with each of the three mental patterns are clearly identified.

**Table 4:** Q-sample statements used for Q-sorting.

Q statement	Resource
1. AI is a transformative technology, but engineers still do not have a clear understanding of what it can do.	Huang (2023) and Huang et al. (2022b)
2. Engineers’ attitudes toward AI depend heavily on how much training and experience they have with it.	Forsythe (1993), Khogali and Mekid (2024) and Buck et al. (2022)
3. Many engineers do not know the differences between AI, machine learning, and deep learning.	Chahal and Gulia (2019) and Tapeh and Naser (2023)
4. Some engineers see AI as a threat to their professional roles.	Abioye et al. (2021) and Raji et al. (2022)
5. A lack of knowledge and clear standards prevents widespread adoption of AI.	ÓhÉigeartaigh et al. (2020) and Hyde et al. (2024)
6. Fear of algorithmic errors and unclear results reduces engineers’ trust in AI.	Zerilli et al. (2022) and Lotfalian Saremi and Bayrak (2021)
7. The complexity of AI algorithms makes them “black box.”	Tschider (2020)
8. High implementation costs and weak IT infrastructure are major barriers to adopting AI.	Shang et al. (2023)
9. The main worries are concerns about data security and accountability for AI-driven decisions.	Farinu (2025) and Alhitmi et al. (2024)
10. Relying too much on AI may weaken engineers’ professional judgment.	Okunola (2025)
11. Intelligent engineers often view AI as an unreliable tool.	Lorge Parnas (1988), Hopgood (2021) and Khaleel et al. (2023)
12. AI helps reduce human errors and saves time and cost.	Hasan et al. (2025) and Elmsellem et al. (2025)
13. AI makes it possible to analyze massive datasets and improve project management efficiency.	Hossain et al. (2024), Wang et al. (2022) and Pshichenko (2024)
14. AI is effective in analyzing structural analysis and predicting how structures will perform during earthquakes.	Al Banna et al. (2020) and Bhatia et al. (2023)
15. AI helps monitor structural cracks, track project progress, and assess material quality.	Chakurkar et al. (2023) and Pala et al. (2025)
16. AI plays a role in soil stability analysis, road alignment design, and tunnel safety evaluations.	Kumar et al. (2026) and Paul et al. (2025)
17. AI improves structural design and offers optimized engineering solutions.	Soori (2024)
18. AI supports risk management and improves safety standards in construction projects.	Usama et al. (2024), Oyetunji et al. (2024) and Abbas (2025)
19. AI helps predict the service life of structural components and supports predictive maintenance.	Ucar et al. (2024) and Farce et al. (2025)
20. AI helps optimize energy use and manage water resources in projects.	Krishnan et al. (2022) and Kamyab et al. (2023)
21. AI improves the quality of technical reporting and transparency in supply chains.	Khan et al. (2022) and Talla (2022)
22. Working with AI and successful real-world projects make engineers more positive toward it.	Prieto (2019)
23. On-the-job training and successful case studies make it easier for engineers to accept AI.	Maity (2019)
24. Support from senior management and collaboration across different disciplines help with AI adoption.	Alamäki (2026) and Cubric (2020)
25. Engineers prefer AI systems that are transparent, explainable, and traceable.	Raji et al. (2022) and Hussain et al. (2021)
26. AI should clearly show uncertainty and confidence levels in its results.	Zhang et al. (2020) and Cau et al. (2023)
27. The ability to customize models and combine different datasets is important to engineers.	Ahmed et al. (2025) and Crawford et al. (2019)
28. A simple user interface and easy-to-understand outputs help increase AI adoption.	Odunuga and Franklin (2024) and Dakulagi et al. (2025)
29. Engineers want AI give recommendation based on engineering principles.	Rafsanjani and Nabizadeh (2023) and Khaleel et al. (2023)
30. AI is increasingly being integrated with other technologies such as IoT and AR.	Alahi et al. (2023)
31. AI should provide recommendations, not make the final decisions.	Wang et al. (2022)

**Table 3:** Total defined variance.

Patterns	Special values			Sum of rotated squares		
	Sum	% Variance	% Cumulative	Sum	% Variance	% Cumulative
1	2/663	26/627	26/627	3/521	35/213	35/213
2	6/604	26/040	52/666	1/984	19/835	55/048
3	1/405	14/047	66/713	1/167	11/665	66/713

**Table 5:** Rotated matrix of factors.

Participants	Patterns		
	3	2	1
P2	−0.143	0.211	0.789
p1	0.029	−0.189	0.745
P3	0.047	−0.089	0.708
P5	−0.169	0.453	0.656
P9	−0.106	0.490	0.538
P10	0.424	0.449	0.505
P6	−0.011	0.812	−0.128
P7	0.200	0.783	0.062
P8	−0.087	0.781	0.103
P11	0.865	0.039	0.118
P4	0.853	−0.044	−0.345

Given that the factor loadings greater than 0.456 =  2.58√32 are considered significant at the 99% confidence level (Brown, 1980), the highlighted factor loadings can be regarded as statistically meaningful. Accordingly, Participants 2, 1, 3, 5, 9, and 10 collectively form Factor One; Participants 8, 6, and 7 collectively form Factor Two; and Participants 11 and 4 constitute Factor Three.

### 4.1 Identification of mental patterns

By calculating the factor score for the three identified groups (mental patterns) and by sorting the factors within each factor group, the statements that received the highest agreement or disagreement in each mental pattern were identified. The results of this analysis are presented in Tables 6–8.

**Table 6:** Defining statements for the Concerned–Hopeful mental pattern (Factor 1).

The most important statement of agreement			The most important statement of disagreement		
Rank	Statements	Score	Statements	Rank	Score
31	A user interface and presenting information in simple language make engineers more willing to accept AI.	1/510	Intelligent engineers often view AI as an unreliable tool.	1	−2.453
30	AI should clearly show uncertainty and confidence levels in its results.	1/335	Some engineers see AI as a threat to their professional roles.	2	−2.262
29	Relying heavily on AI may weaken engineering judgment.	1/266	AI is effective in analyzing structural analysis and predicting how structures will perform during earthquakes.	3	−1.385
28	Data security and responsibility for AI-driven decisions are main issues.	1/257			
27	AI Engineers prefer systems that are transparent, explainable, and traceable.	1/194			
26	AI is increasingly being integrated with other technologies such as IoT and AR.	1/048			
25	Engineers want AI to give recommendations based on engineering principles.	0/892			

**Table 7:** Defining statements for the Black-Box mental pattern (Factor 2).

The most important statement of agreement			The most important statement of disagreement		
Rank	Statements	Scores	Rank	Statements	Scores
31	The complexity of AI algorithms makes them “black box.”	2/619	1	High implementation costs and weak IT infrastructure are major barriers to adopting AI.	−3.152
30	Working with AI and successful real-world projects make engineers more positive toward it.	1/140	2	Intelligent engineers often view AI as an unreliable tool.	−1.493
29	Many engineers do not know the differences between AI, machine learning, and deep learning.	1/013	3	The main worries are concerns about data security and accountability for AI-driven decisions.	−1.46
28	AI is a transformative technology, but engineers still do not have a clear understanding of what it can do.	0/914			

**Table 8:** Defining statements for the Result-Oriented mental pattern (Factor 3).

The most important statement of agreement			The most important statement of disagreement		
Rank	Statements	Scores	Rank	Statement	Scores
31	AI makes it possible to analyze massive datasets and improve project management efficiency.	1/251	1	Engineers’ attitudes toward AI depend heavily on how much training and experience they have with it.	−2.305
30	AI systems should enable human interaction and allow engineers to provide feedback.	1/177	2	AI helps predict the service life of structural components and supports predictive maintenance.	−2.035
29	AI helps reduce human errors and saves time and cost.	0/771	3	AI is a transformative technology, but engineers still do not have a clear understanding of what it can do.	−1.827
28	AI improves the quality of technical reporting and transparency in supply chains.	0/753	4	AI supports risk management and improves safety standards in construction projects.	−1.808
27	Fear of algorithmic errors and unclear results reduces engineers’ trust in AI.	0/660			

In this mindset, the following statements received higher scores:

“A visual user interface and presentation of information in simple words increase the acceptance of artificial intelligence.”

“AI must specify uncertainties and confidence levels in its results.”

“Overreliance on AI may weaken engineering judgment.”

“Concerns about data security and accountability of AI decisions are among the main issues.”

“Engineers prefer AI systems to be transparent, explainable, and traceable.”

“The combination of AI with other technologies such as IoT and AR is expanding.”

“Engineers expect AI to provide its recommendations based on engineering principles.”

“A lack of adequate knowledge and clear standards prevents the widespread adoption of AI.”

In contrast, the statements “Experienced engineers consider AI to be an unreliable tool,” “Some engineers see AI as a threat to their professional standing,” and “AI is effective in analyzing structural behavior and predicting seismic performance” received lower scores.

Therefore, based on the statements with the highest ratings, this first perspective was named the ‘Concerned-Hopeful’ mindset.

In this mindset, the statements “The complexity of AI algorithms has turned them into a ‘black box’,” “Experience working with AI and successful real-world projects make engineers more positive toward it,” “Many engineers do not know the difference between AI, machine learning, and deep learning,” and “AI is a transformative technology, but engineers do not have a clear understanding of its capabilities,” receive higher scores.

In contrast, the statements “High implementation costs and the lack of IT infrastructure are key barriers,” Experienced engineers consider AI to be an unreliable tool,” and “Concerns about data security and accountability of AI-driven decisions are major issues” receive lower scores.

Therefore, based on the statements that received the highest ratings, the second viewpoint is named the Black Box Mindset.

In this mindset, the statements “AI enables big-data analysis and optimization of project management,” “AI systems should allow human interaction and feedback,” “AI helps reduce human error and saves time and cost,” “AI improves the quality of technical reporting and transparency in the supply chain,” and “Fear of algorithmic error and lack of transparency reduces engineers’ trust” received high scores. On the other hand, the statements “Engineers’ attitudes toward AI depend on their training and experience,” “AI helps predict the service life of structural components and supports predictive maintenance,” “AI is a transformative technology, but engineers do not have a clear understanding of its capabilities,” and “AI is effective in risk management and improving safety standards in projects” received lower scores. Therefore, based on the highest-scoring statements, the third mental pattern was labeled the Results-Oriented Mindset.

## 5 Discussion

Overall, the results of this typology indicate that civil engineers do not have a unified perspective on artificial intelligence. There are three viewpoints: Concerned-Optimistic, Black Box, and, Result-Oriented. The findings of this study, which identified three distinct mental patterns among civil engineers regarding AI applications, not only introduce the core cognitive thoughts in this field but also align meaningfully with existing research. These patterns reflect a range of technical, organizational, and psychological concerns. Based on the typology of mental models identified among civil engineers, the findings show that their professional trust in artificial intelligence systems depends not only on computational accuracy but also on transparency, explainability, and the traceability of decision-making.

The anxious-optimistic mental model, in which engineers simultaneously recognize the potential of AI and express concerns about its implications, directly aligns with concerns highlighted in studies by Krampell et al. (2020). Although civil engineers are concerned about the loss of their professional identity due to the rise of AI, more specialized analysis and application of data with AI are important to them. The findings confirm that this tension represents a central cognitive model that requires special attention in educational and organizational planning. Professional engineering organizations should design formal workshops and protocols to educate engineers on how to interact with, evaluate, and validate AI data. Engineers should learn how to use explainability tools to identify questionable recommendations and critically challenge them when necessary. Also, Civil engineering departments should add topics such as AI ethics, explainability, and big data management into their curricula. The goal is to educate future engineers from the outset with the understanding that technical accuracy alone is insufficient, and that algorithmic accountability is equally essential. Educational and professional frameworks should reinforce the view that AI is a tool designed to assist engineering expertise, not replace it; therefore, professional judgment should always take precedence over system outputs.

On the other hand, the black box mindset emphasizes engineers’ need for transparency, explainability, and trust in AI algorithm results, which clearly overlaps with technical and ethical concerns documented in the scientific literature (Chen et al., 2025; Krampell et al., 2020). This mindset suggests that, especially in civil engineering fields involving safety and high accuracy, AI remains a significant challenge until it becomes an explainable “white box.” AI developers should not focus solely on improving model accuracy, but instead emphasize designing “glass box” models or integrating local explainability techniques.

In contrast, the outcome-oriented paradigm is likely to focus on efficiency, economic benefits, and rapid return on investment (ROI), which is closely aligned with managerial and organizational perspectives in the literature (Shang et al., 2023). This view suggests that these engineers adopt a managerial/business-oriented perspective that focuses on “profit and efficiency.” According to this view, AI-based software should accompany its recommendations with meaningful technical justifications that are understandable to practicing engineers. Rather than reporting an optimal numerical result, the system should make clear which regulatory requirements (such as national building codes) or physical principles (e.g., stress reduction at a critical location) were met in achieving that result. It is also recommended that AI systems deployed in safety-critical applications, such as bridge monitoring or risk management, should provide full traceability of their inputs. Regulatory authorities (such as the Roads, Housing and Urban Development Organization) should set requirements in future regulations and standards governing the use of AI in formal structural calculations and designs, requiring professional certification. These requirements should include standard justification reports that are formally documented in the project’s technical files.

## 6 Conclusion

The study emphasizes the importance of the absence of a single perspective on AI. The differences between identified viewpoints, emphasis on profit in the Result-Oriented pattern versus focus on accuracy in the Black Box, pose a critical challenge for project managers: the necessity of managing conflicting organizational and technical perspectives simultaneously to ensure successful AI adoption in the construction industry.

This research scientifically demonstrates that explainability is not a luxury feature but a vital engineering requirement. The mental patterns of civil engineers indicate that in the absence of the ability to understand and trace AI decisions, professional trust will not form, and the use of AI in critical projects will remain limited.

Engineers’ demands across various patterns for “feedback incorporation,” “ethical protocols,” and “human oversight” highlight the urgent need for human-in-the-loop models (systems that actively involve engineers as the final responsible specialists in AI decision-making processes).

In conclusion, this study not only revealed existing mental patterns but also provides a framework for formulating local requirements for algorithmic transparency in civil engineering. These should be considered in educational curricula, technical regulations, and software development standards.

This study also faced several limitations. First, the comprehensiveness of the statement set represents a challenge. Despite substantial efforts to capture technical, ethical, economic, and particularly explainability-related dimensions of artificial intelligence, the breadth and rapidly evolving nature of the field mean that some niche or secondary perspectives within the civil engineering community may not have been fully represented. Second, the inherent complexity of artificial intelligence posed a limitation. Concepts such as explainability and traceability are technically sophisticated, and some respondents may have encountered difficulties in meaningfully differentiating between statements due to limited familiarity with their underlying technical details.

## Data Availability

The raw data supporting the conclusions of this article will be made available by the authors, without undue reservation.
